# Cellulose nanofibrils prepared by gentle drying methods reveal the limits of helium ion microscopy imaging

**DOI:** 10.1039/c9ra01447k

**Published:** 2019-05-20

**Authors:** Annika E. Ketola, Miika Leppänen, Tuomas Turpeinen, Petri Papponen, Anders Strand, Anna Sundberg, Kai Arstila, Elias Retulainen

**Affiliations:** VTT Technical Research Centre of Finland Ltd P. O. Box 1603 FI-40101 Jyväskylä Finland elias.retulainen@vtt.fi; University of Jyväskylä, Nanoscience Centre, Department of Physics and Department of Biological and Environmental Science FI-40014 Jyväskylä Finland; Åbo Akademi University, Johan Gadolin Process Chemistry Centre Porthansgatan 3 FI-20500 Åbo/Turku Finland

## Abstract

TEMPO-oxidized cellulose nanofibrils (TCNFs) have unique properties, which can be utilised in many application fields from printed electronics to packaging. Visual characterisation of TCNFs has been commonly performed using Scanning Electron Microscopy (SEM). However, a novel imaging technique, Helium Ion Microscopy (HIM), offers benefits over SEM, including higher resolution and the possibility of imaging non-conductive samples uncoated. HIM has not been widely utilized so far, and in this study the capability of HIM for imaging of TCNFs was evaluated. Freeze drying and critical point drying (CPD) techniques were applied to preserve the open fibril structure of the gel-like TCNFs. Both drying methods worked well, but CPD performed better resulting in the specific surface area of 386 m^2^ g^−1^ when compared to 172 m^2^ g^−1^ and 42 m^2^ g^−1^ of freeze dried samples frozen in propane and nitrogen, respectively. HIM imaging of TCNFs was successful but high magnification imaging was challenging because the ion beam tended to degrade the TCNFs. The effect of the imaging parameters on the degradation was studied and an ion dose as low as 0.9 ion per nm^2^ was required to prevent the damage. This study points out the differences between the gentle drying methods of TCNFs and demonstrates beam damage during imaging like none previously reported with HIM. The results can be utilized in future studies of cellulose or other biological materials as there is a growing interest for both the HIM technique and bio-based materials.

## Abstract 2

TEMPO-oxidized cellulose nanofibrils (TCNFs) have unique properties, which can be utilised in many application fields from printed electronics to packaging.

## Graphical abstract

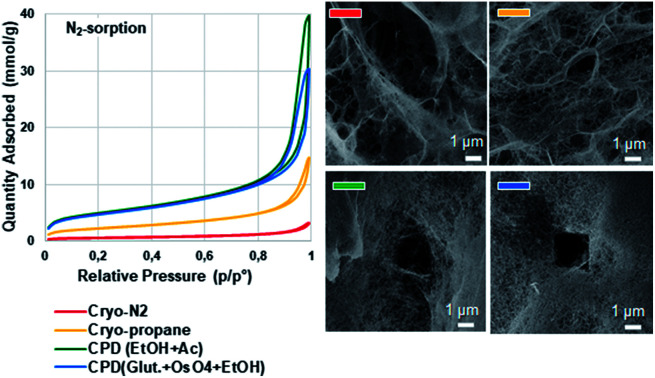

## 1 Introduction

Cellulose nanofibrils (CNFs) have been under intensive investigation due to their unique properties, such as high tensile strength, large specific surface area, rheology and tendency for film formation.^1^ CNFs can be prepared from natural cellulose wood fibres by mechanical and chemical treatments,^2^ which makes them a biodegradable and renewable material. Various different application fields exist for CNFs ranging from paper, composites and food additives to bio-film material for printed electronics or packaging, biomedical applications and aerogels.^3–8^ Thus, proper characterisation of CNFs is important for quality control and safety assessment.^9^ The nanoscale size, the branched fibril structure and the high water content make the characterisation of CNFs challenging and demand sophisticated techniques. Scanning electron microscopy (SEM) is often used for characterisation of the morphology of CNFs and fibril dimensions in the mm to nm scale.^9–13^ Transmission electron microscopy (TEM) is the most powerful imaging technique and can be utilized for detailed nanoscale evaluation of single fibril dimensions of CNFs.^9,14–17^ SEM usually requires a conductive coating before imaging and the TEM method is limited to thin samples like single fibrils, and because of that, the new imaging method scanning helium ion microscopy (HIM) has aroused interest during recent years.

In the HIM method, a focused helium ion beam releases secondary electrons (SE) from the sample surface and creates an image in a similar way to SEM.^18–20^ Because of the HIM single atom source, the probe is smaller compared to SEM, which together with the smaller excited surface volume makes higher imaging resolution possible.^18,21^ Another advantage is that non-conductive samples do not need coating because the charging can be compensated for with an electron flood gun. HIM is widely utilized as a nanofabrication tool because helium ion beams can modify surfaces in the nanoscale by ion sputtering or implantation;^22–24^ however, it is reported that in imaging applications the beam damage for organic samples is negligible.^20,25,26^

Thus far the HIM-imaging method has been used for cellulose-based materials usually with low to intermediate resolutions. Li *et al.* (2016)^27^ used HIM for successful imaging of paper-like composites of activated carbon and mechanically fibrillated CNFs whereas Torvinen *et al.* (2017)^4^ studied kaolin—CNF composites of different CNF-types. Virtanen *et al.* (2018)^28^ studied mechanically fibrillated CNF-aerogels, and HIM had a good depth of view for the freeze-dried CNF-aerogel when compared to SEM. The only study with high-resolution imaging of cellulose-based materials is by Postek *et al.* (2011)^21^ where cellulose nanocrystals on mica were imaged. Because of that, the high-resolution imaging capabilities of HIM for cellulosic materials are still unclear.

Vacuum based imaging methods require dry samples and in order to obtain images that represent the nanocellulose structure in the wet-state, a gentle drying procedure is necessary. Improper drying will result in the collapse of the pores and coalescence of fibrils.^29^ This can be prevented, for example, by using cryofixing, in which the sample is rapidly frozen in a cryoliquid, followed by drying of the frozen sample under a vacuum, where the ice sublimates into a gas without collapsing the structure. Liquid nitrogen (LN_2_) is commonly used for freezing of various samples but is known to be affected by the Leidenfrost-effect where the boiling liquid forms an insulating gas layer between the sample and the coolant. This delays the freezing process, giving time for unwanted structural changes caused by ice crystal formation. With liquid propane (LPGS), used near its freezing point, this effect is reduced.^15^ Another method for the preservation of the wet structure of the material is the critical point drying (CPD), where the collapse of the sample structure is prevented by passing the liquid–gas interphase in the drying by replacing the solvent with the supercritical fluid which is then turned into gas.^30^

Gentle drying of CNFs from water using cryofixing or CPD results an aerogel-like materials with large surface area.^11,31,32^ Porous aerogels are often used as an insulators or adsorbents, but unlike brittle silica or carbon, native CNF-aerogels have been shown to possess mechanical toughness, flexibility and softness.^11,33–35^ CNF-aerogel studies have involved CNFs of different types, drying techniques and applications, including conductive enzymatic-CNF aerogels dried with cryo-LPGS,^33^ insulating and transparent TEMPO-oxidized liquid crystalline CNF aerogels dried with CPD,^34^ magnetic bacterial-CNF aerogels dried with freeze drying,^35^ soft enzymatic-CNF and TCNF aerogels dried with CPD^11^ and hydrophobic mechanically fibrillated CNF aerogels dried with freeze drying.^31^ The effect of drying techniques on CNFs structure^31,36^ and properties^37,38^ has been demonstrated showing unambiguously how the CPD drying is able to preserve the open fibril structure of CNFs better than freeze drying. TCNF aerogels have been also shown to have higher specific surface area (SSA)^39^ than bacterial-CNF when dried with the same technique.^11^ A high SSA indicates that the original open fibril structure has been well preserved during and SSA as high as 480 m^2^ g^−1^ has been reported for TEMPO-oxidized CNFs after CPD.^40^ Also freeze drying can achieve relatively high (100–300 m^2^ g^−1^) SSA values for CNFs depending on the applied procedure.^10–12,32,41^

The objective of this study was to find the optimal preparation methods for wet TEMPO-oxidized CNFs (TCNFs) in order to preserve its fine fibril structure and evaluate the suitability of HIM for imaging the porous CNF-material with high resolution. TCNFs were selected for the work over other CNF-types as they have well-characterized fine and homogeneous structure, large specific surface area and high charge;^14^ thus, they can be considered to be the most delicate structures to reveal the convenience of the methods. Also, TCNFs alone have not been imaged with HIM and the effect of different drying techniques on TCNF aerogel structures has not been evaluated before.

TCNFs were dried with four different drying procedures including cryofixing with LN_2_ or LPGS followed by freeze drying and solvent exchange followed by CPD including two different solvent exchange procedures. The first CPD procedure involved sample fixation with glutaraldehyde (GA) and osmium tetroxide (OsO_4_) and dehydration with ethanol (EtOH) before CPD. The second procedure involved only dehydration using EtOH and acetone (AE) prior to CPD. Nitrogen (N_2_)-sorption and BET-analysis were used to determine the SSA of the TCNFs samples in order to quantify the differences between the drying methods. The results are expected to give useful information for the future studies of delicate bio-based structures with HIM.

## 2 Experimental

### 2.1. Materials

#### 2.1.1. Chemicals

Propane (class 2, UN 1965: 95% propane and 5% butane) and liquefied nitrogen (LN_2_) was purchased from AGA Gas Ab, Lidingö, Sweden. Acetone (AE, ≥99.9%) was obtained from Sigma-Aldrich, Darmstadt, Germany. Sodium cacodylate (NaCac, R1104, Agar Scientific, Stansted, UK) was acquired as a powder and a 0.4 M buffer stock-solution was prepared using ultrapure-water. Glutaraldehyde (GA, 25%-solution for electron microscopy) was obtained from Merck (Darmstadt, Germany) and used as a 2%-solution in 0.1 M NaCac-buffer solution (pH 7.4). Osmium tetroxide (OsO_4_, Electron Microscopy Sciences, Hatfield, USA) was a 4%-solution, which was diluted to a 2%-stock solution with ultrapure-water. Ethanol (EtOH, absolute AA, Etax) was obtained from Altia Oyj, Rajamäki, Finland.

#### 2.1.2. TEMPO-oxidized CNFs

TCNFs were prepared from never-dried birch kraft pulp by TEMPO-mediated oxidation and fluidization. TEMPO-mediated oxidation was performed according to a previously described procedure,^2,14^ in which fibres were first suspended in a water solution of TEMPO and sodium bromide. Then, NaClO solution was added to the suspension (5 mmol to 15 mmol per gram of fibres) and the pH was adjusted to 10 at room temperature with NaOH. The reaction was considered complete when the pH remained stable. After oxidation, the fibres were washed thoroughly with deionized water followed by treatment with a microfluidizer M7115-30 (2 passes). The carboxylic content of TCNFs, determined by conductometric titration, was approximately 1.0 mmol g^−1^ of dry CNFs. Detailed characterisation of these particular TCNFs can be found elsewhere.^42^

### 2.2. Methods

#### 2.2.1. Gentle drying of TCNFs using cryofixing

The TCNFs were cryofixed in LN_2_ (cryo-LN_2_) and in LPGS (cryo-LPGS). LPGS was prepared by liquefying propane gas using LN_2_ and cooled until its freezing temperature (−189 °C) was nearly reached. A drop of TCNF-gel (1.08% [w/w]) was placed on a TEM grid (300 mesh) and immediately plunged in LN_2_ (approximately −196 °C) or in LPGS. The cryofixed samples were placed on a LN_2_ cooled metal plate and dried in a freeze drier at −50 °C under vacuum (Christ LOC-1m) over night. The dried samples were kept in a desiccator until HIM imaging or BET-analysis was carried out.

#### 2.2.2. Gentle drying of TCNFs using solvent exchange and critical point drying (CPD)

Two different methods for solvent exchange prior to CPD of TCNFs were used. Solvent exchange with GA, OsO_4_ and EtOH was performed by first attaching a drop of TCNF-gel (1.08% [w/w]) to a glass coverslip with epoxy-based glue. Samples were placed in a 24-microtiter plate containing a fixative (2% GA in a 0.1 M NaCac buffer, pH 7.4) and incubated for 4 h. The samples were then washed with 0.1 M NaCac buffer twice and incubated with 1% OsO_4_ in 0.1 M NaCac for 30 min, after which the washing with 0.1 M NaCac was repeated three times. After fixation with GA and OsO_4_, the samples were dehydrated to EtOH by using a series of steps with increasing EtOH concentration: 50, 70, 90, 95 and 2 × 99.5%. The dwell time in each step was 30 min, and the final step took place overnight.

Solvent exchange with EtOH and AE was done first by dehydrating drops of TCNF-gel in an EtOH stepwise, as explained earlier. After the last step in 99.5% EtOH the sample was placed in AE overnight. CPD (Leica CPD 300, University of Jyväskylä, Finland) was the last step from EtOH or AE to the ambient conditions. The CPD programme included 16 exchange cycles of CO_2_ at medium speed (speed value 5) without stirring. Slow speed was used for gas filling, heating, and venting steps. The dried samples were attached to metal stubs using carbon tape and kept in a desiccator until HIM-imaging.

#### 2.2.3. Helium ion microscopy (HIM)

HIM (Zeiss Orion Nanofab, University of Jyväskylä Nanoscience Centre, Finland) was used for imaging the dried TCNFs. Acceleration voltage of 30 to 35 kV with aperture 10 μm was used resulting to an ion current of 0.1–0.3 pA. Image size 1024 × 1024 pixels, line averaging between 4 to 16 lines, dwell time 0.5 or 1.0 μs and working distance approximately 9 mm were used as the imaging parameters. All samples were studied without metal coating, and the electron flood gun with 750 eV energy was used to neutralize the sample charging. Fibril dimensions were estimated from HIM images by using ImageJ software (ImageJ freeware, USA). The scale bar of the images was used to turn the software pixels into nanometers and the fibrils width were collected from different spots of the image so that rough estimation of different fibril widths could be done.

#### 2.2.4. N_2_-sorption

The SSA of the TCNFs was determined using the Brunauer–Emmett–Teller (BET) method.^39^ Approximately 70 mg of dried TCNFs was prepared for the analysis. TCNFs were dried using the procedures described before (Sections 2.2.1 and 2.2.2) with small modifications. In cryofixing, the sample volume was increased to approximately 0.2–0.3 g so that the structure was still able to freeze rapidly during the plunge-freezing in LN2 or in LPGS. In solvent exchange, the sample volume was increased to approximately 0.4 g (a large drop), incubation time in GA-solution was for overnight and in OsO_4_ for 60 min. The dwell time in EtOH series was 60 min (the final step took place overnight) and after the last step in 99.5% EtOH the samples were placed in AE over two nights. Prolonged incubation times were conducted to ensure proper replacement of water in the structure. Samples were kept in a desiccator until N_2_-sorption measurement.

The dried TCNFs was weighed in sample flasks and de-gassed in a vacuum at 110 °C for 30 min. After that the temperature was raised to 125 °C for 4 h. Finally, the temperature was increased to 150 °C for 15 min. The samples were then placed in a N_2_-sorption device (Micromeritics 3Flex Version 4.04, VTT Espoo) and the adsorption data was collected at −196 °C by adjusting the relative nitrogen pressure from 0 to 0.99 and back. The Barrett–Joyner–Halenda (BJH) theory was used to calculate the average equivalent pore size of the TCNFs based on the N_2_-sorption isotherms.^43^ The model is based on an assumption of spherically shaped pores, which is not the case in a fibril network system. Thus, the obtained values were mainly used as relative guidelines when comparing the samples. The average equivalent fibril diameter (*d*) was also estimated from the SSA values using eqn (1). The density of cellulose was assumed to be 1600 kg m^−3^ (ref. 44 and 45) and fibrils were assumed to be infinitely long rods with a cylindrical cross section.1
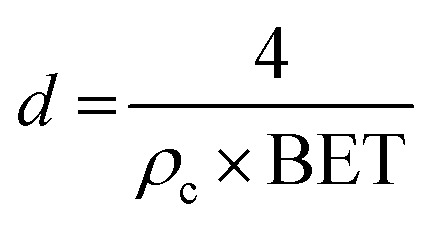
*d* = fibril diameter, *ρ*_c_ = cellulose density and BET = BET specific surface area.

## 3 Results and discussion

### 3.1. HIM-imaging of TCNF-gel

**Fig. 1:**
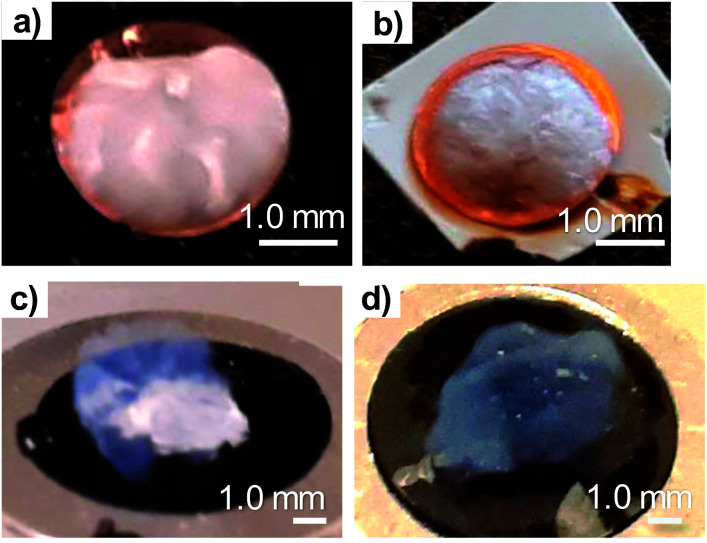
Dried TCNFs attached on black carbon tape ready for HIM imaging. (a) cryo-LN2, (b) cryo-LPGS (white epoxy glue shows underneath the slightly transparent sample), (c) CPD (EtOH, AE) (d) CPD (GA, OsO_4_, EtOH).

Both cryofixation and CPD preserved well the shape of the TCNFs. The samples could be handled without being fractured and not any clear shrinkage of the samples were observed. Cryofixed samples were white in colour and resembled dry polystyrene foam in appearance (Fig. 1a and b). CPD dried samples were light blue, transparent and resembled fine-structured cottonwool (Fig. 1c and d). Blue colour could be a result of the Rayleigh scattering in the material with small length scales, previously observed also with TEMPO-oxidized liquid crystalline CNF-aerogels dried by using CPD^34^ and silica-based aerogels.^46^

**Fig. 2:**
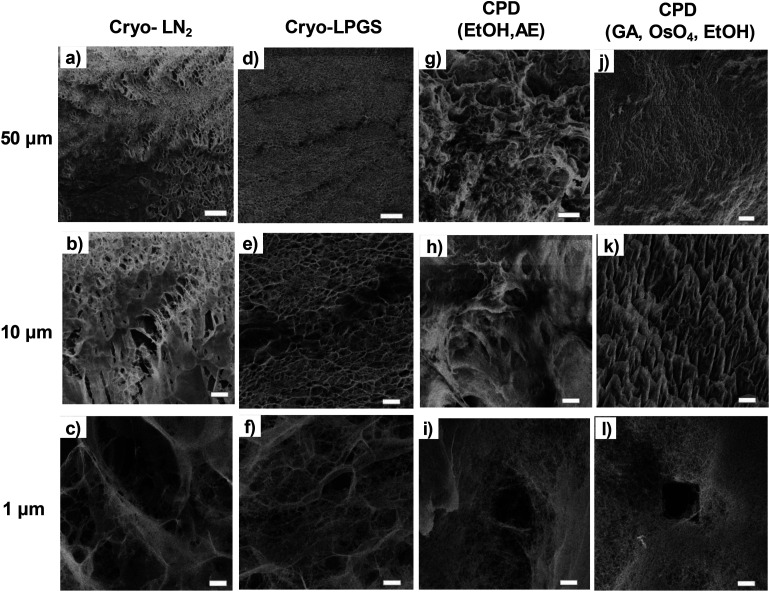
HIM-images of dried TCNFs with different magnifications. (a), (b), (c) cryo-LN_2_, (d), (e), (f) cryo-LPGS, (g), (h), (i) CPD (EtOH, AE) and (j), (k), (l) CPD (GA, OsO_4_, EtOH). Top row FoV 400 μm, middle row FoV 100 μm and bottom row FoV 10 μm. Fig. 1i and l show clearly the holes caused by the ion beam.

**Fig. 3:**
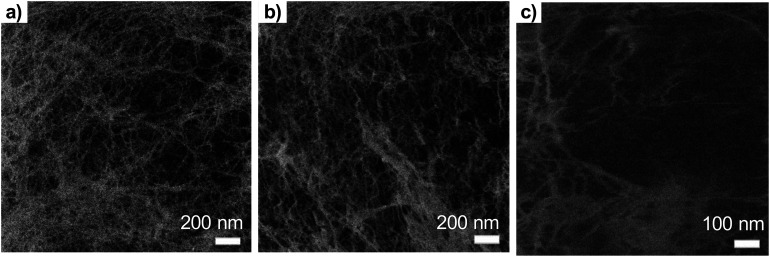
The effect of the ion dose to the beam damage on the TCNFs (a) ion dose 4 × 10^13^ ions per cm^2^ (b) 9 × 10^13^ ions per cm^2^ (c) 4 × 10^14^ ions per cm^2^ (eqn (2)).

Low magnification HIM images of dried TCNFs with a field of view (FoV) of 400 and 100 μm presents dense and wavy surfaces (Fig. 2a–k). Waviness was most probably caused by sample handling and liquid fluctuations during the drying. In order to see the differences between the actual fibril structures, a closer investigation with higher magnification (FoV 10 μm) was needed. High magnification HIM images (Fig. 1c, f, i and l) show that TCNF-surfaces consisted of a very fine fibrillar material. The CPD-dried samples resembled each other also at higher magnification. Rough estimation of fibril dimensions from the HIM images showed approximately 20 nm-wide fibrils, or fibril bundles, in all samples. Accurate statistical analysis of fibril dimensions and distributions was not possible as the sample degraded during imaging or pictures were too noisy (Fig. 3). TCNFs are found to consist of single fibrils of 3–4 nm in width^14^ but fibrils of those dimensions could not be distinguished here. There were also much thicker 100 nm wide fibril bundles in the cryofixed samples. Similar structural differences between freeze dried and CPD dried commercial-CNF^36^ and enzymatic-CNF^15^ have been observed before with SEM.

The formation of clear TCNF-films was more severe in cryo-LN_2_ samples than cryo-LPGS samples. The heterogeneous structure and the film formation indicated that there was ice crystal formation during cryofixing of the sample that pushed the fine material to the edges of the ice crystals.^10,16,40,47^ The CPD-dried samples showed a more homogenous microstructure. In addition to the small size, TCNFs have high negative charge and high specific surface area,^14^ meaning that they bind a lot of water in their structure. Removal of the water in such a way that the fine fibril structure remains open is challenging and was not fully achieved with the used cryofix-methods.

Detection of single fibrils turned out to be challenging because of the sample degradation during the imaging. Especially, the CPD-dried TCNFs were sensitive at higher magnifications. Imaging, as in Fig. 2c, f, i and l, was conducted by first using the 2 μm FoV to focus a beam and align the flood gun and then the actual image was taken with 10 μm FoV. Because of the focusing step, a clear hole was formed to the center of the image as can be seen in Fig. 2i and l. Smaller FoV had a higher ion dose per area and to confirm that the effect was dose based, the imaging was done to the fresh areas with different doses by adjusting the amount of averaging. The total ion dose was calculated with eqn (2):2
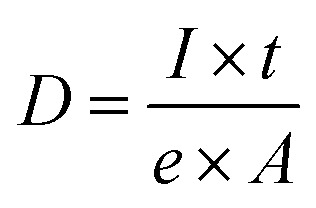
*D* = areal dose, *I* = ion current, *t* = pixel dwell time and *A* = pixel area.

With dose of 4.9 × 10^13^ ions per cm^2^ some fibrillar network could be resolved, but the noise level was quite high, which caused the graininess of the image (Fig. 3a). When the dose was doubled (Fig. 3b), some deformations of the fibril structure was detected already. Interestingly, this dose was under the theoretical limit of the sub-nanometer imaging because there were 0.9 ions per nm^2^. When tenfold dose was used (Fig. 3c) the structure of the fibril network was collapsed leaving some individual fibrils drift over the imaging area.

Degradation of the bio-based materials during the HIM-imaging has not been described in the literature previously. For example, Joens *et al.* (2013)^26^ demonstrated how biological organisms can be imaged with high magnifications without damage and in the study by the Leppänen *et al.* (2017)^22^ the dose 1.1 × 10^16^ ions per cm^2^ was used to image the dried agar gel network of 10 nm fibrils with no clear damage. Comparison of the ion doses among the literature is challenging because usually not all the imaging parameters are listed. Fox *et al.* (2013)^48^ studied a graphene flakes with HIM and Raman spectroscopy and found that dose of 10^17^ ions per cm^2^ was required for proper edge contrast to obtain sub-nanometer resolution and 5 × 10^14^ ions per cm^2^ already caused significant damage to graphene lattice. Livengood *et al.* (2009)^49^ studied the defect formation in the silicon and copper by HIM and found that with over 5 × 10^15^ ions per cm^2^, subsurface lattice dislocations are found from the TEM cross-sections. These materials are quite different compared to the polymeric CNFs and the direct comparison is not possible.

CNFs consist of cellulose chain bundles with alternating amorphous and crystalline regions having intermolecular hydrogen bonding between the chains on the crystalline part.^50,51^ The amorphous regions of the network can be considered the weakest points of the structure. Most probably, the ionization of the cellulose by the ion beam, especially in the amorphous regions collapses the structure. Actually, single ion can cause several ionizations because it is known that secondary electron yield of helium ranges from 3 to 10 depending on the material^18^ Crystalline CNCs, in which the amorphous regions are no longer present, have been imaged with HIM without similar degradation.^21,28^ However, the quality and magnification of the images and sample preparation methods cannot be directly compared to this work.

Cellulose-based materials have been found to be a highly sensitive also to the electron beams and the imaging of the single cellulose nanofibril or cellulose nanocrystal is challenging with TEM.^52^ More specifically, a critical dose where the diffraction from the crystalline part of nanocellulose has been halved as the sign of destruction has been found to be about 6 × 10^15^ electrons per cm^2^. This is about 10-times more than the helium ion dose causing the collapse of the fibrillar network in our experiment.

**Fig. 4:**
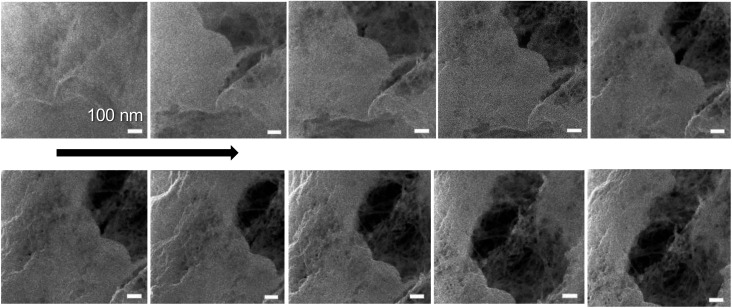
Time series of the same area of TCNFs dried with CPD (GA, OsO_4_, EtOH) showing the effect of beam damage (or ion milling) on the sample surface within seconds when using the 0.3 pA ion current. Total imaging time 80 seconds (8 s per image). Imaged using a 16-line average with FoV 1 μm. Scale bar is 100 nm for all the images.

An interesting application of the beam damage is demonstrated in Fig. 4, which shows the time series over the sample area with 0.3 pA ion current with total imaging time of couple of minutes. The overall structure in the first figure was quite plain with no individual fibrils observed. When the same area was imaged multiple times, a hole started to form in the right-upper corner as a loosely bound film-like fibril layer on top of the sample milled away revealing the underlying fibril network. This, so-called “unwrapping” property of the ion beam could be generally utilized to detect different materials based on the milling rate. The image area shifts a little bit upward and right during the imaging, which was also a common finding. The fibril network underneath was most likely collapsing and resulted in an overall change of shape. In principle, helium ions can penetrate tens of μm deep with this acceleration voltage, which means that also the structure underneath the surface can be damaged.

Traditionally OsO_4_ has been used to reduce sample charging during imaging with SEM.^53,54^ In the current study the fixation with glutaraldehyde and OsO_4_ (Fig. 1l) did not protect the TCNFs against degradation. Atomic layer deposition or chemical vapour deposition of titanium oxide^16,31^ or sputtering of a Pt or Au/Pd layer on the sample surface could provide a protective layer for fibrils and enable imaging with high magnifications; however, metal sputtering can distort the fibril dimensions^26,55,56^ and possibly damage the finer fibril structure.

### 3.2. Specific surface area (SSA) of TCNFs

**Fig. 5:**
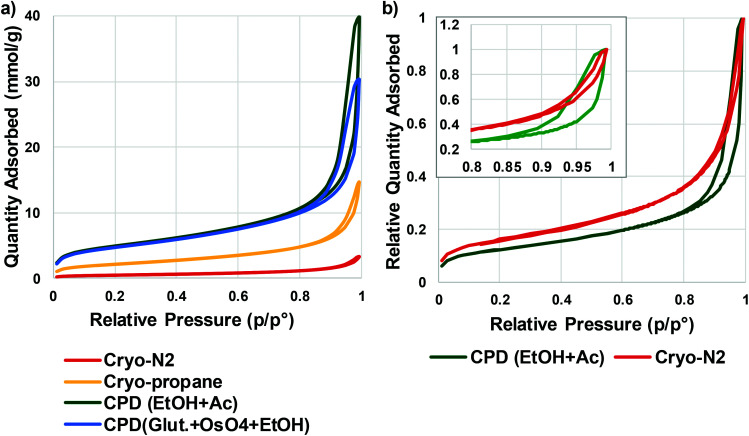
(a) N_2_-sorption isotherms of TCNFs dried with gentle drying methods (b) relative N_2_-sorption isotherms of cryo-N_2_ and CPD (EtOH, Ac) dried TCNFs.

**Fig. 6:**
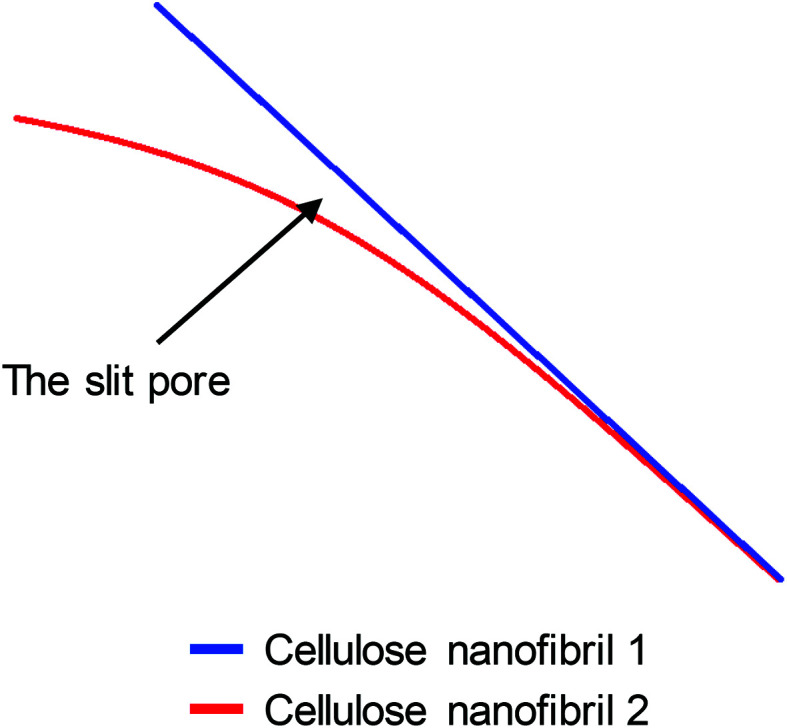
A sketch of possible configuration of a slit-shaped pore between two cellulose nanofibrils.

**Fig. 7:**
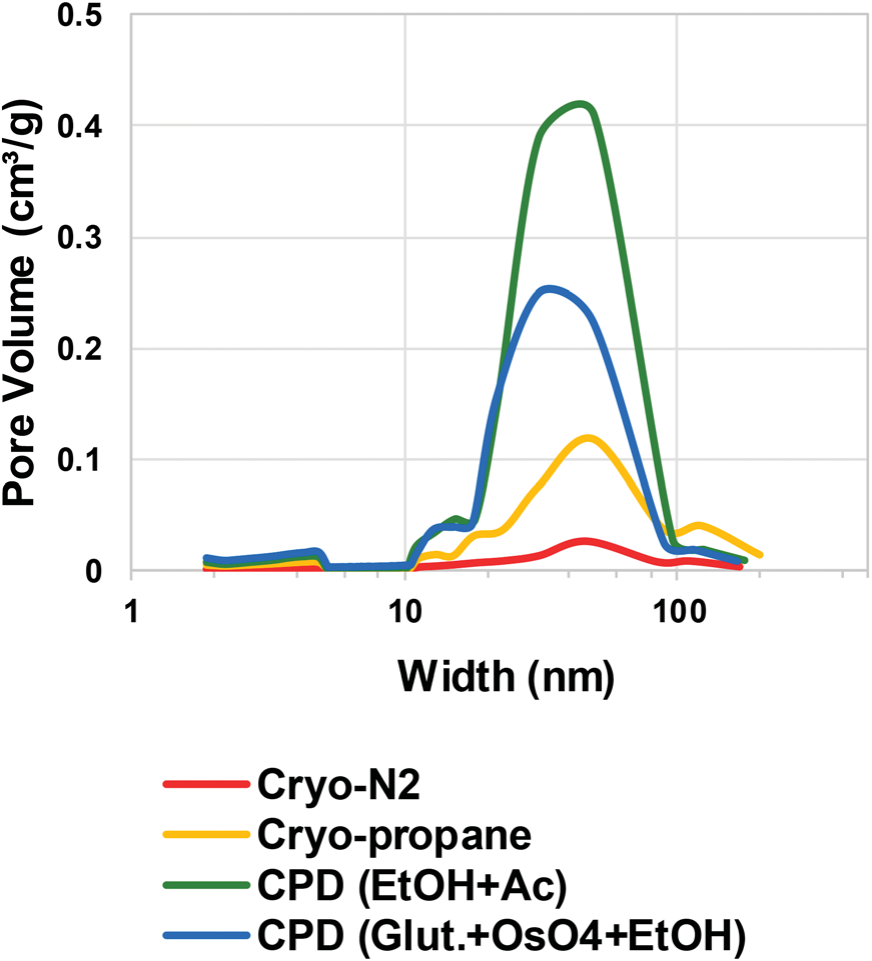
Estimated pore size distribution obtained using BET method of the TCNFs dried with different gentle drying methods. Average pore width (nm) on the *x*-axis at logarithmic scale and pore volume (cm^3^ g^−1^) on the *y*-axis.

Quantitative evaluation of the differences between the gentle drying methods of TCNFs was done by using BET-analysis, which determines the specific surface area (SSA) of a material that is accessible to nitrogen. In the case of TCNFs, the higher the SSA, the more open the structure and the less coalescence of fibrils has taken place during drying. All of the samples had a type IV N_2_-sorption isotherm curves with a type H3 hysteresis loop (Fig. 5), which means monolayer-multilayer adsorption of nitrogen on mesoporous structure with pore widths between 2–50 nm.^57,58^ This hysteresis type typically indicates that the structure was formed of aggregates of platy particles with slit-shaped pores (Fig. 6).^11,32,57–60^ This slit-shape possibly makes pores prone to collapse during drying, as surfaces want to minimize their energies by binding to each other and the closer they are, the stronger are the surface energies leading to collapse.^61^ Samples dried with cryo-LN_2_ did not show any hysteresis loop in the N_2_-sorption isotherms. When the adsorbed quantities were plotted as relative values (Fig. 5b; the adsorbed quantity divided by the highest detected adsorption), a small hysteresis loop could be detected. There was a steep increase in the isotherms after the relative pressure of 0.82, and because capillary condensation in mesopores occurs at the higher pressure values, the result indicates that most of the pores were larger in size. This was also seen in the pore size distribution (Fig. 7.) of the samples where most of the pores were in the mesopore and micropore range (15–100 nm). Over 50 nm pores or pores under 2 nm cannot be detected accurately by BET, and for this reason, it is probably not the most suitable method for fibrillated materials like TCNFs, but the results were still useful for comparing differences between the samples in the current study.

Table 1 shows the mean values of SSA, equivalent pore size, pore volume, and fibril diameter values. The Barrett–Joyner–Halenda (BJH) method was used to calculate the pore size distributions of the samples. The CPD-dried samples adsorbed more nitrogen than cryofixed samples and had higher SSA, equivalent pore size and pore volume. CPD (EtOH, Ac) samples had the largest SSA (386 m^2^ g^−1^), equivalent pore size (14.3 μm) and pore volume (1.4 cm^3^ g^−1^). The equivalent pore size was similar to the rest of the samples (around 11 μm) but the pore volume was significantly lower for cryofixed samples than for CPD samples. Less than 2% of all pores in all samples were of micro scale, and the rest of the pores were mesopores or larger (Table 1 and Fig. 7). This could mean that even though the volume of the pores in cryofixed samples was lower than in CPD samples, the pore structure was similar. According to the results, CPD drying was able to prevent the coalescence of the fibril structure better than cryofixing; however, most of the micropores in the structure were not preserved or could not be detected. As high SSA values as 500–600 m^2^ g^−1^ have been reported for TEMPO-oxidized liquid crystalline CNF-aerogels dried by using CPD and EtOH–solvent exchange.^34^ The liquid crystalline arrangement achieved by acid-treatment could have promoted the structure stability, which was also observed as high toughness of the dried material. CPD has been reported to be a promising drying method also for TCNF-nanopapers, yielding SSA values of 480 m^2^ g^−1^ measured by N_2_-sorption^40^ and TCNFs dehydrated with EtOH and AE before CPD showed higher nitrogen adsorption and SSA than samples fixed with GA and OsO_4_ before dehydration in EtOH. The last AE step could be more beneficial for CPD as the CO_2_-gas is more miscible with AE than with EtOH.^62^ In electron microscopy, GA in combination with OsO_4_ is a commonly used protein fixative of both plant and animal samples.^53,63^ On the other hand, it has been observed to be more efficient in preservation of internal plant structures than surfaces,^63^ and did not provide any significant support for cellulose-fibril structures against the ion beam damage in the current study.

**Table 1:** Mean values of the SSA, equivalent pore size and pore volume of TCNFs dried with different gentle drying methods

	BET surface area	Pore size^b^	Fibril diameter	Pore volume^a^	Micropore volume <2 nm
	m^2^ g^−1^	nm	nm	cm^3^ g^−1^	cm^3^ g^−1^
Cryo-N_2_	41.8	10.7	59.8	0.11	0.0012
Cryo-LPGS	171.8	11.6	14.6	0.50	0.0036
CPD (EtOH, Ac)	385.7	14.3	6.5	1.38	0.0193
CPD (GA, OsO_4_, EtOH)	375.0	11.2	6.7	1.05	0.0176

a Single point desorption total pore volume of pores less than 193.5 nm width at *p*/*p*_o_ = 0.990.

b Desorption average pore diameter (4*V*/*A* by BET).

Again, cryo-LPGS samples showed higher nitrogen adsorption and SSA (172 m^2^ g^−1^) than cryo-LN_2_ samples (SSA 42 m^2^ g^−1^). LN_2_ is known to suffer from the Leidenfrost-effect, and the low SSA was probably a result of the coalescence of fibrils by the ice crystal formation. The corresponding results of drying efficiency of cellulose microfibrils (CMF) with CPD, LN_2_ and LPGS have been previously reported.^15^ Sehaqui *et al.* (2011)^11^ reported a SSA of 150–280 m^2^ g^−1^ for TCNFs dried from water using solvent exchange to EtOH and *tert*-butanol and cryofixing with LN_2_. Exchanging the water inside the material to a solvent with low surface tension has been shown to increase the SSA also for regenerated cellulose (160–190 m^2^ g^−1^).^10^ Thus, it could be possible to increase the SSA of cryo-LPGS samples by first conducting a solvent-exchange of the samples, like it is done with samples prepared for CPD. On the other hand, if the target is to image TCNFs as they appear in water, where certain charges and interactions between fibrils occur, the solvent-exchange from water to a non-polar solvent could also change these interactions and the surface structure. Thus, it is not obvious that the higher SSA value of the solvent-exchanged samples really describes the sample structure in aqueous conditions.

The calculated equivalent fibril diameters of TCNFs were approximately 7 nm, 15 nm and 60 nm for CPD, cryo-LPGS and cryo-LN_2_ dried samples, respectively. Large fibril diameters of cryofixed TCNFs can be explained by the fibril agglomeration during drying. Multilayer adsorption of nitrogen can increase the detected surface area, which decreases the calculated fibril diameters, and the actual diameters were most likely larger. The BET results (Table 1) supported the observations from the HIM images of TCNFs (Fig. 2). CPD resulted more homogeneous and finer fibril structure, with less intensive film formation compared to cryofixing. As mentioned before, the smallest fibrils observed from the HIM images were approximately 20 nm in width (Fig. 2c, f, i and l), which was significantly larger than the width estimates from the BET-analysis. This could be due to beam damage during the imaging, making the smallest fibrils disappear or due to the multilayer adsorption of nitrogen in the BET-analysis that affected the SSA calculations. In addition, the HIM-images show only the surfaces of the samples and do not represent the whole structure.

Quantitative differences between different drying methods was not obtained by microscopy, and for that N_2_-sorption and BET-analysis were needed. Based on these results (Fig. 2 and Table 1), the solvent exchange in EtOH/AE combined with CPD drying is the most preferred method, and also involves less hazardous chemicals and liquid exchange steps than treatment with GA/OsO_4_/EtOH. Solvent exchange with AE combined with CPD is found to result in a high SSA also with the fibres;^64^ however, is worth to keep in mind that solvent exchange could modify the interactions between the fibrils that occur in water. TCNFs have added carboxyl groups on the cellulose chain, which increases their hydrogen bonding ability in water. When water is exchanged to the less polar media the hydrogen bonding is hindered and can be responsible for more open fibril structure in the dried material. Cryofixing in LPGS surpasses LN_2_ in SSA values, but cryofixing in LN_2_ is more simple and faster than cryofixing in LPGS. In order to select a suitable drying method for TCNFs one needs to consider if a highly preserved structure is necessary, and how much time and effort is practical to use.

## 4 Conclusions

The suitability of HIM for imaging the porous TCNF-aerogels with high resolution was evaluated and different aerogel preparation methods using gentle drying were compared. High-resolution HIM-imaging of TCNFs was compromised by the dose-related damage as not described before with ion beams. Further research is needed about ion beam induced damage on the organic materials to have reliable imaging methods in the future. Comparison of the different gentle drying methods showed that all methods preserved the wet structure of TCNFs at some degree. CPD was considered to be the best method for drying delicate samples with SSA of 386 m^2^ g^−1^. Cryo-LPGS provided moderate result with SSA of 172 m^2^ g^−1^, but SSA for cryo-LN_2_ was only 42 m^2^ g^−1^, and should be carefully considered if detailed surface structures of wet cellulose fibril materials are studied. Sample handing procedure in the preparation phase seemed also to affect the large-scale structures of the sample, but clear systematic differences between the samples was seen only in nanoscale.

## Conflicts of interest

There are no conflicts to declare.

## Supplementary Material
